# Multifocal Neuroarthropathy of the Knee and Foot Induced by Physical Training of the Lower Extremities in a Patient With Latent Autoimmune Diabetes in Adults

**DOI:** 10.7759/cureus.28163

**Published:** 2022-08-19

**Authors:** Chika Kojima, Tatsuhito Himeno, Machiko Akao, Hideki Kamiya, Jiro Nakamura

**Affiliations:** 1 Internal Medicine, Owariasahi Clinic, Owariasahi, JPN; 2 Division of Diabetes, Department of Internal Medicine, Aichi Medical University School of Medicine, Nagakute, JPN; 3 Department of Orthopedic Surgery, Aichi Medical University School of Medicine, Nagakute, JPN; 4 Department of Innovative Diabetes Therapy, Aichi Medical University School of Medicine, Nagakute, JPN

**Keywords:** diabetic polyneuropathy, total knee arthroplasty, physical training, latent autoimmune diabetes, neuroarthropathy, charcot knee

## Abstract

Charcot neuroarthropathy is a progressive arthropathy associated with neuropathy. In patients with diabetes, Charcot neuroarthropathy mostly affects the foot. In the present case, we encountered a rare presentation of Charcot neuroarthropathy of the knee and foot in a patient with latent autoimmune diabetes in adults. The patient, who may have developed the disease as a result of inappropriate physical exercise, was treated with total knee arthroplasty.

## Introduction

Charcot neuroarthropathy is a progressive arthropathy accompanied by a background of sensory neuropathy [1]. Charcot neuroarthropathy in the setting of diabetic polyneuropathy (DPN) is recognized as a serious complication in patients with diabetes. DPN is a symmetrical sensorimotor neuropathy that progresses mainly at the distal portion of the lower extremities [2]. Symptomatic DPN is found in about 20-50% of patients with diabetes [3-5] and affects approximately 50% of patients with diabetes during their lifetime [6]. However, it is reported that around 75% of patients with diabetes have some nerve conduction abnormalities at the onset of diabetes [7]. Given DPN progresses from the distal portion of the lower extremities, diabetic Charcot neuroarthropathy is common in the foot joints [8]. Although diabetic neuroarthropathy rarely affects the knee, it is increasingly being reported [9]. Here, we report a rare case of Charcot neuroarthropathy in the knee and foot involving a 38-year-old male with diabetes who developed severe sensory dysfunction and deformity in the left knee and right foot.

## Case presentation

A 38-year-old male was referred to our hospital complaining of edema in his left knee and right foot. He had no regular health checkups. At the age of 33, he was diagnosed with type 2 diabetes. However, he did not continue treatment after that. At that time, he was aware of thermal hypoesthesia in bilateral lower limbs up to the level of the distal thighs. At the age of 36, as he was found to be positive for anti-glutamic acid decarboxylase antibody, insulin therapy was initiated. Thereafter, the patient began training using a leg extension machine. About six months before, the right ankle began to swell, and about four months later, the left knee also began to swell. The patient was seen in a clinic because of a deformity of the knee while swimming. At that time, he walked using two crutches due to the difficulty in walking caused by the deformity of the knee. The patient had no history of cardiovascular events, physical trauma, participation in extreme sports, alcohol abuse, or smoking. The patient was admitted to the hospital for further study and treatment. His height was 157 cm and his weight was 65 kg. Physical examination revealed redness, swelling, and warmth in his left knee and right foot. As the patient developed hyperalgesia, it was difficult to assess deep tendon reflexes and vibration perception at the lower extremities. On admission to the hospital, serological tests including vitamins, creatinine kinase, thyroid function, C-reactive protein, anti-nuclear antibodies, anti-cyclic citrullinated peptide antibody, matrix metalloproteinase 3, and rheumatoid factor were all unremarkable (Table 1).

**Table 1:** Hematological and serological results.

Laboratory test	Value	Reference range and units
Hematology		
White blood cells	9.2	5.0–8.0 × 10^3^/μL
Neutrophils	65.8	42.6–58.9%
Lymphocytes	24.3	30.3–40.5%
Monocytes	8.3	3.3–6.2%
Eosinophils	1.4	0.0–4.5%
Basophils	0.2	0.0–1.9%
Red blood cells	3.66	4.50–5.10 × 10^6^/μL
Hemoglobin	11.1	13.9–16.0 g/dL
Hematocrit	33.3	41.4–49.2%
Mean corpuscular volume	91.0	81.0–97.0 fL
Platelets	29.5	18.0-35.0 × 10^4^/μL
Serology		
Total protein	7.1	6.7–8.3 g/dL
Albumin	4.2	4.0–5.0 g/dL
Total bilirubin	0.89	0.3–1.2 mg/dL
Aspartate aminotransferase	26	13–33 IU/L
Alanine aminotransferase	15	6–30 IU/L
Alkaline phosphatase	397	115–359 U/L
γ-Glutamyl transpeptidase	19	10–47 IU/L
Lactate dehydrogenase	349	119–229 U/L
Urea	20.2	8–22 mg/dL
Creatinine	0.58	0.60–1.10 mg/dL
Estimated glomerular filtration rate	124	>60.0 mL/minute/1.73m^2^
Uric acid	6.2	3.6–7.0 mg/dL
Sodium	141	138–146 mEq/L
Potassium	4.5	3.6–4.9 mEq/L
Chloride	104	99–109 mEq/L
Calcium	9.2	8.7–10.3 mg/dL
Inorganic phosphorus	4.0	2.5–4.7 mg/dL
Hemoglobin A1c	5.7%	4.6–6.2%
Casual blood glucose	164	NA, mg/dL
C-peptide	0.5	1.1–3.3 ng/mL
Triglycerides	51	30–149 mg/dL
Total cholesterol	196	128–219 mg/dL
High-density lipoprotein-cholesterol	46	40–96 mg/dL
Low-density lipoprotein-cholesterol	95	70–139 mg/dL
Vitamin B1	48	24–66 ng/mL
Vitamin B12	774	180–914 pg/mL
Folic acid	>=22.0	>4.0 ng/mL
Anti-glutamic acid decarboxylase antibody	9.8	<1.5 U/mL
Anti-insulin antibody	35.6	<0.4 U/mL
Thyroid stimulation hormone	1.462	0.350–4.940 µIU/mL
Free thyroxine	1.14	0.70–1.48 ng/dL
Parathyroid hormone, intact	36	10–65 pg/mL
Tartrate-resistant acid phosphatase-5b	1130	170–590 mU/dL
Undercaroxylated osteocalcin	6.16	<4.5 ng/mL
C-reactive protein	0.06	<0.30 mg/dL
Rheumatoid factor	<3.0	<15.0 IU/mL
Anti-cyclic citrullinated peptide antibody	<0.6	<4.5 U/mL
Matrix metalloproteinase 3	78	37–121 ng/mL

Serological tests for syphilis including rapid plasma reagin and *Treponema pallidum* latex agglutination were negative. The urine albumin/creatinine ratio was 189.4 mg/gCr. Hemoglobin A1c was 5.7%. Anti-glutamic acid decarboxylase antibody and anti-insulin antibody were 9.8 U/mL and 35.6 pg/mL, respectively. Bone metabolism-related tests were significant: tartrate-resistant acid phosphatase-5b was 1,130 mU/dL (reference range: 170-590 mU/dL for males) and undercarboxylated osteocalcin was 6.16 ng/mL (reference range: <4.5 ng/mL). Nerve conduction studies and electromyography are depicted in Tables 2, 3.

**Table 2:** Motor nerve conduction studies. NR = no response

Nerve	Velocity (m/s)	Latency (ms)	Amplitude (mV)
Right median	45.9	3.66	12.66
Left median	47.6	3.48	6.97
Right ulnar	41.7	3.51	3.08
Left ulnar	49.0	3.33	12.02
Right tibial	NR	NR	NR
Left tibial	39.1	2.67	16.52

**Table 3:** Sensory nerve conduction studies. NR = no response

Nerve	Velocity (m/s)	Latency (ms)	Amplitude (mV)
Right median	38.8	3.22	8.4
Left median	47.2	2.86	14.4
Right ulnar	36.0	2.92	11.0
Left ulnar	37.6	2.66	10.2
Right sural	NR	NR	NR
Left sural	NR	NR	NR

**Figure 1:**
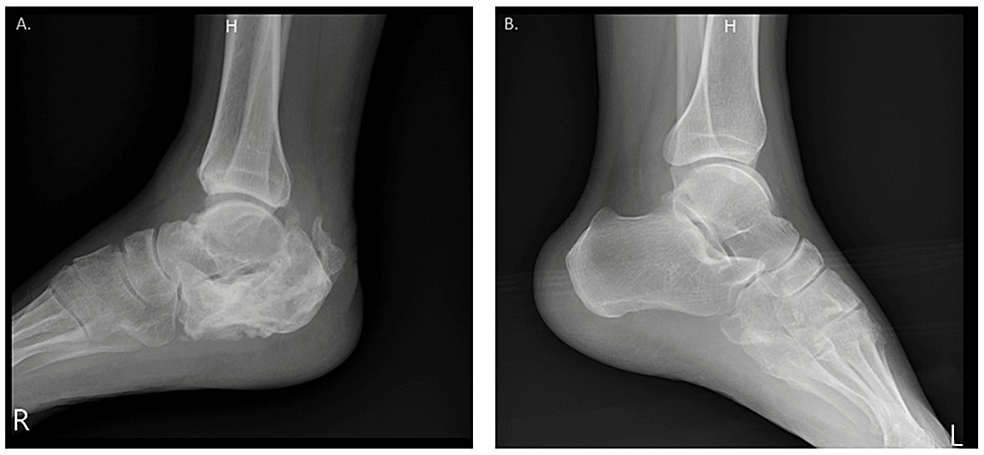
The lateral views of the foot and ankle. A: Fragmentation of the calcaneal bone with associated bony erosions and disorganization in the right foot. B: Normal shape and alignment of the left foot.

Results showed absent bilateral sural and right tibial responses. Bilateral median and right ulnar motor distal latencies were within normal limits with normal compound motor action potential (CMAP) amplitudes and reduced motor nerve conduction velocity (MNCV). Left tibial motor distal latency was within normal limits with normal CMAP and reduced MNCV. Sensory nerve conduction velocity (SNCV) in the right median and bilateral ulnar nerves were reduced. The ankle-brachial pressure indices were within the normal range (right leg 1.22, left leg 1.19). His daily medications included insulin glulisine, insulin detemir, amlodipine, hydrochlorothiazide, and losartan. Radiographs of the right foot demonstrated fragmentation of the calcaneal bone with associated bony erosions and disorganization, consistent with Charcot neuroarthropathy (Figure 1).

**Figure 2:**
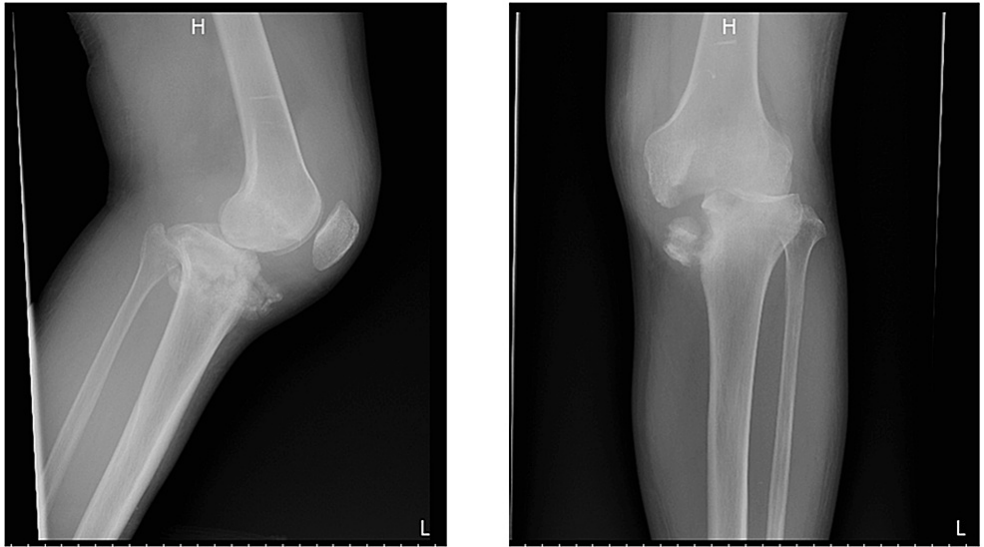
Radiographs of the left knee on admission. Lateral (left) and anteroposterior (right) views show a comminuted fracture of the medial tibial condyle.

In the left knee, there was a comminuted fracture of the medial tibial condyle and narrowing of the knee joint space with bony erosion (Figure 2).

**Figure 3:**
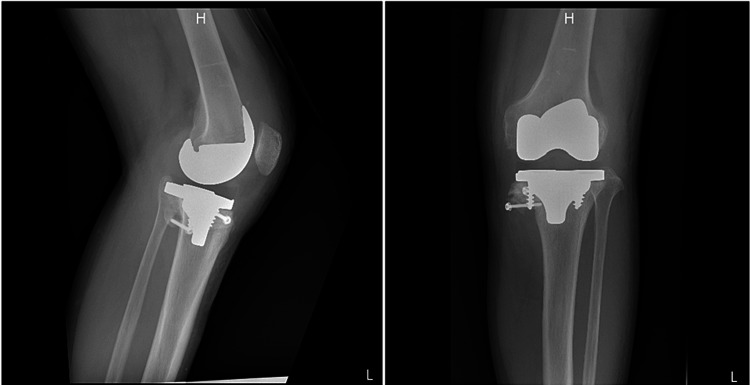
Postoperative radiographs of the left knee. Lateral (left) and anteroposterior (right) views show the restored alignment of the left knee after total knee arthroplasty.

Magnetic resonance imaging of the spinal cord showed no findings of syringomyelia. We diagnosed him with diabetic Charcot neuroarthropathy of the right foot and left knee. Left total knee arthroplasty (TKA) was performed without complication (Figure 3).

After physiotherapy, he was capable of walking without crutches at the time of discharge. The neuroarthropathy in the right foot was conservatively managed with offloading, resulting in no progression at the current five-year mark. After the TKA, the function of the left knee has not deteriorated.

## Discussion

Charcot neuroarthropathy is the non-infectious destruction of joints in patients with peripheral sensory neuropathy. The pathogenesis of Charcot neuroarthropathy has been much debated, that is, neuro-vascular theory and neuro-traumatic theory [8]. Among the hypotheses of the pathogenesis, the neuro-traumatic theory could be applicable in the current case. The first step of the theory is that impaired afferent innervation results in decreased proprioception and deep pain sensation. Consequently, when the insensate joints are subjected to repetitive trauma, the joints can be fractured and deformed. In this case, the patient who had severe sensory neuropathy had a habit of exercising using a leg extension machine. It can be inferred that, as a result, his insensate knee joint was repetitively injured and eventually destructed. Additionally, as the serological findings indicated the increase in osteoclastic bone resorption, the increase in osteoclastic activity might have precipitated the onset and progression of the arthropathy [10].

Charcot neuroarthropathy of the knee is rare. According to the scoping review article reported by Lu et al., 40 of 212 patients with neuroarthropathy of the knee had diabetes, second only to syphilis in 85 patients [9]. In this report, being overweight, the most important risk factor for other knee diseases, such as osteoarthritis, was not a risk factor for neuroarthropathy, as the mean body mass index (BMI) was 23.51 kg/m^2^. In this case, the BMI was 26.4, making it unlikely that being overweight was a significant risk factor for the development of the disease. Additionally, in the report, the mean duration from the onset of subjective symptoms to the initial presentation was 50.5 months, which pointed out the importance of early diagnosis. Fortunately, our patient was diagnosed two months after the onset of knee symptoms and immediately treated. Regarding treatment, there is no universal treatment algorithm. Treatment options include conservative treatment using knee braces, arthrodesis, and TKA. In the 21st century, TKA has been performed more frequently and has been shown to improve quality of life with fewer complications. In this case, TKA was involved as the primary management and produced the uncomplicated result. Approximately 10% of patients with diabetic Charcot neuroarthropathy have bilateral lesions [11]. Although the present patient also has bilateral neuroarthropathy of the lower extremities, fortunately, no progression has been observed at the five-year mark. However, as the pathogenesis of this disease remains to be elucidated, the course of this case should continue to be monitored.

## Conclusions

Although it is generally recognized that diabetic Charcot neuroarthropathy affects the foot joints, in rare cases, this disease can occur in other joints including the knee. When a patient has severe sensory neuropathy, as in this case, the patient should be advised to avoid repetitive exercises that induce mechanical stress on joints of lower extremities, such as the knee.
